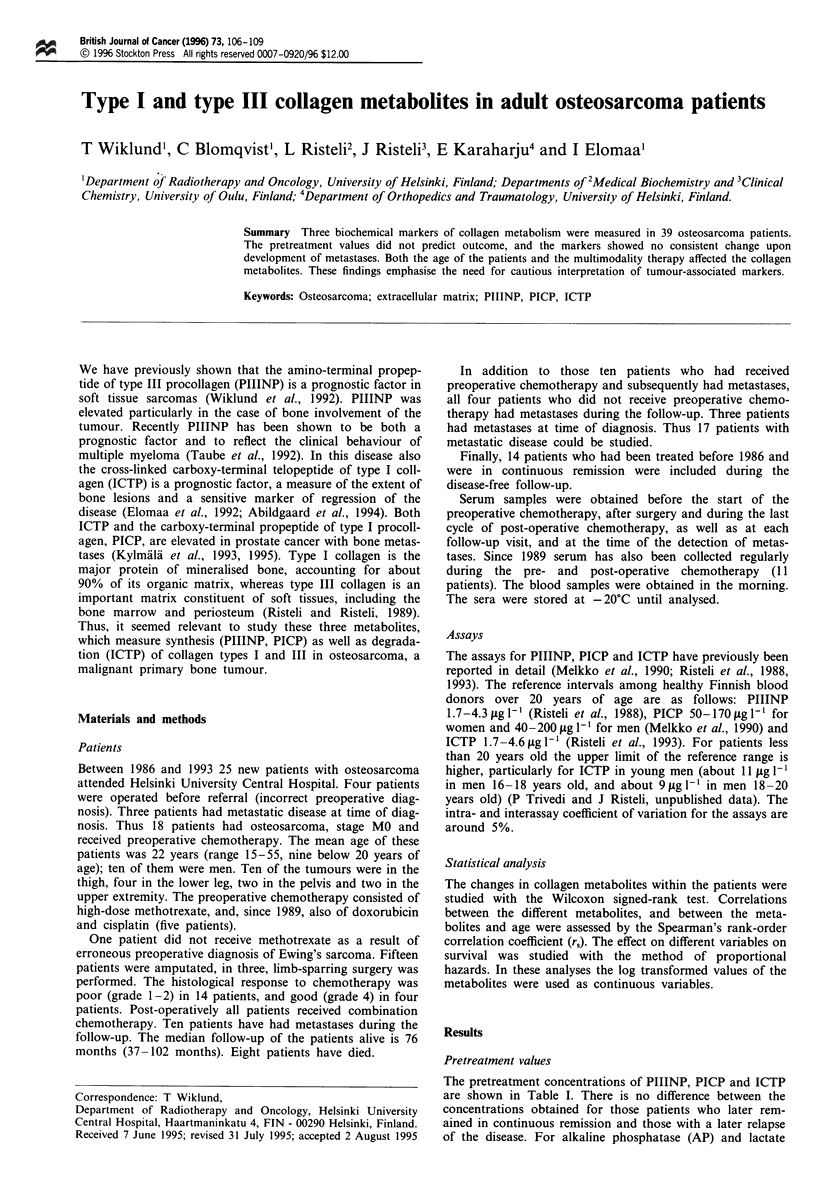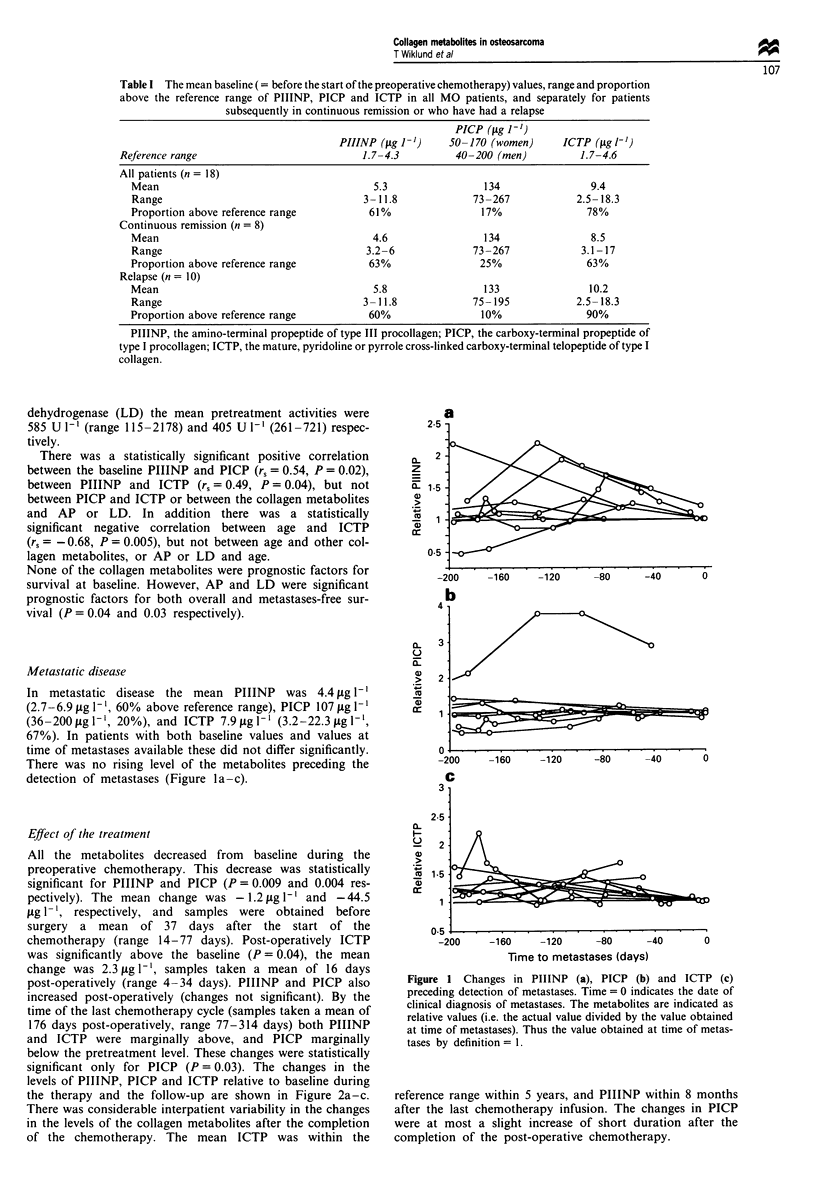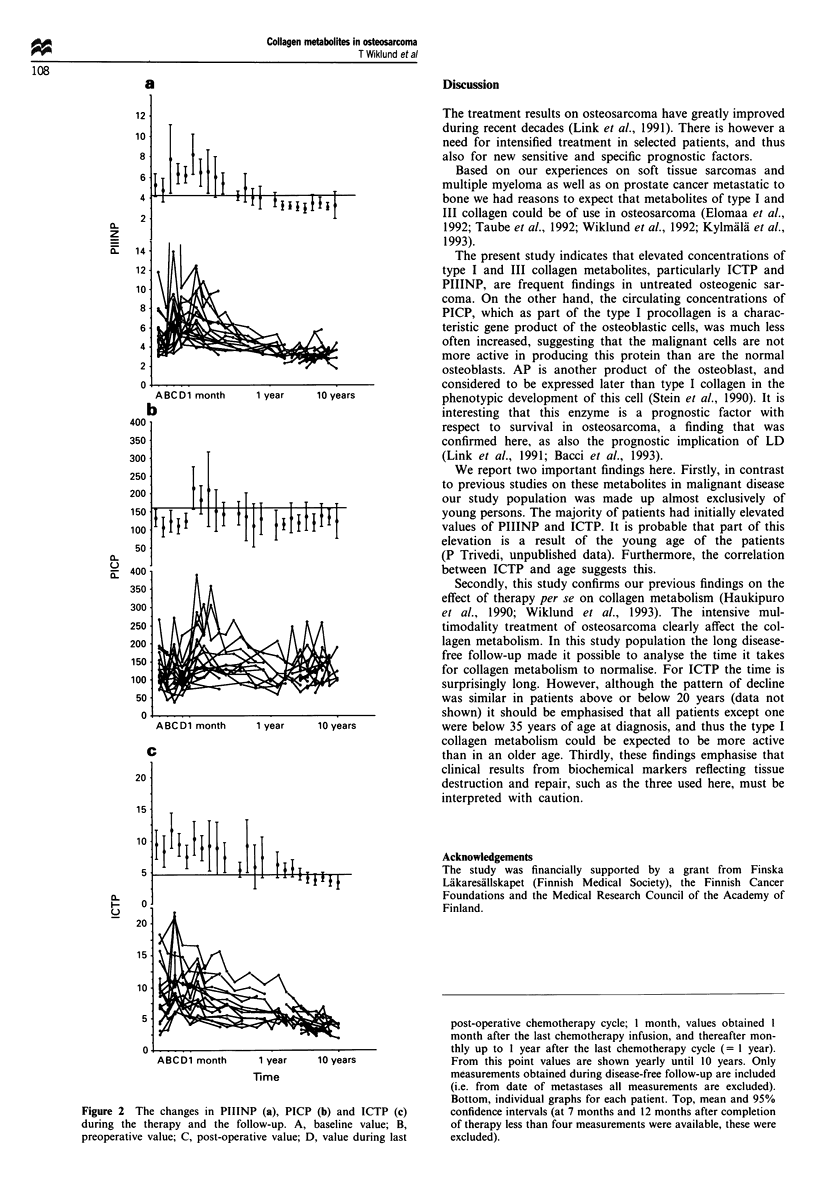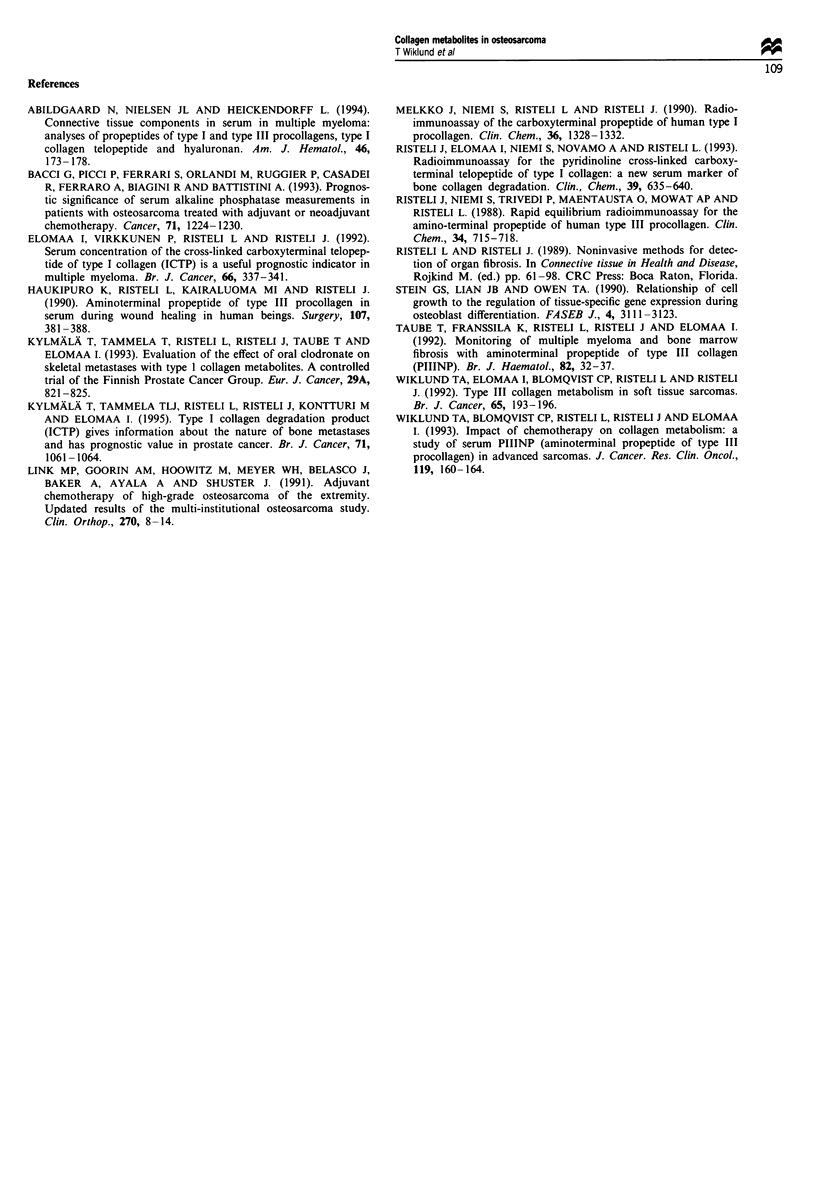# Type I and type III collagen metabolites in adult osteosarcoma patients.

**DOI:** 10.1038/bjc.1996.19

**Published:** 1996-01

**Authors:** T. Wiklund, C. Blomqvist, L. Risteli, J. Risteli, E. Karaharju, I. Elomaa

**Affiliations:** Department of Radiotherapy and Oncology, University of Helsinki, Finland.

## Abstract

Three biochemical markers of collagen metabolism were measured in 39 osteosarcoma patients. The pretreatment values did not predict outcome, and the markers showed no consistent change upon development of metastases. Both the age of the patients and the multimodality therapy affected the collagen metabolites. These findings emphasise the need for cautious interpretation of tumour-associated markers.


British Journal of Cancer (1996) 73, 106–109
© 1996 Stockton Press All rights reserved 0007–0920/96 $12.00

### Type I and type III collagen metabolites in adult osteosarcoma patients

T Wiklund[1], C Blomqvist[1], L Risteli[2], J Risteli[3], E Karaharju[4] and I Elomaa[1]

[1]*Department of Radiotherapy and Oncology, University of Helsinki, Finland; Departments of* [2]*Medical Biochemistry and* [3]*Clinical Chemistry, University of Oulu, Finland;* [4]*Department of Orthopedics and Traumatology, University of Helsinki, Finland.*

**Summary** Three biochemical markers of collagen metabolism were measured in 39 osteosarcoma patients. The pretreatment values did not predict outcome, and the markers showed no consistent change upon development of metastases. Both the age of the patients and the multimodality therapy affected the collagen metabolites. These findings emphasise the need for cautious interpretation of tumour-associated markers.

**Keywords:** Osteosarcoma; extracellular matrix; PIIINP, PICP, ICTP

We have previously shown that the amino-terminal propeptide of type III procollagen (PIIINP) is a prognostic factor in soft tissue sarcomas (Wiklund *et al.*, 1992). PIIINP was elevated particularly in the case of bone involvement of the tumour. Recently PIIINP has been shown to be both a prognostic factor and to reflect the clinical behaviour of multiple myeloma (Taube *et al.*, 1992). In this disease also the cross-linked carboxy-terminal telopeptide of type I collagen (ICTP) is a prognostic factor, a measure of the extent of bone lesions and a sensitive marker of regression of the disease (Elomaa *et al.*, 1992; Abildgaard *et al.*, 1994). Both ICTP and the carboxy-terminal propeptide of type I procollagen, PICP, are elevated in prostate cancer with bone metastases (Kylmälä *et al.*, 1993, 1995). Type I collagen is the major protein of mineralised bone, accounting for about 90% of its organic matrix, whereas type III collagen is an important matrix constituent of soft tissues, including the bone marrow and periosteum (Risteli and Risteli, 1989). Thus, it seemed relevant to study these three metabolites, which measure synthesis (PIIINP, PICP) as well as degradation (ICTP) of collagen types I and III in osteosarcoma, a malignant primary bone tumour.

### Materials and methods

### *Patients*

Between 1986 and 1993 25 new patients with osteosarcoma attended Helsinki University Central Hospital. Four patients were operated before referral (incorrect preoperative diagnosis). Three patients had metastatic disease at time of diagnosis. Thus 18 patients had osteosarcoma, stage M0 and received preoperative chemotherapy. The mean age of these patients was 22 years (range 15–55, nine below 20 years of age); ten of them were men. Ten of the tumours were in the thigh, four in the lower leg, two in the pelvis and two in the upper extremity. The preoperative chemotherapy consisted of high-dose methotrexate, and, since 1989, also of doxorubicin and cisplatin (five patients).

One patient did not receive methotrexate as a result of erroneous preoperative diagnosis of Ewing's sarcoma. Fifteen patients were amputated, in three, limb-sparring surgery was performed. The histological response to chemotherapy was poor (grade 1–2) in 14 patients, and good (grade 4) in four patients. Post-operatively all patients received combination chemotherapy. Ten patients have had metastases during the follow-up. The median follow-up of the patients alive is 76 months (37–102 months). Eight patients have died.

Correspondence: T Wiklund,
Department of Radiotherapy and Oncology, Helsinki University Central Hospital, Haartmaninkatu 4, FIN - 00290 Helsinki, Finland.
Received 7 June 1995; revised 31 July 1995; accepted 2 August 1995

In addition to those ten patients who had received preoperative chemotherapy and subsequently had metastases, all four patients who did not receive preoperative chemotherapy had metastases during the follow-up. Three patients had metastases at time of diagnosis. Thus 17 patients with metastatic disease could be studied.

Finally, 14 patients who had been treated before 1986 and were in continuous remission were included during the disease-free follow-up.

Serum samples were obtained before the start of the preoperative chemotherapy, after surgery and during the last cycle of post-operative chemotherapy, as well as at each follow-up visit, and at the time of the detection of metastases. Since 1989 serum has also been collected regularly during the pre- and post-operative chemotherapy (11 patients). The blood samples were obtained in the morning. The sera were stored at $-20°C$ until analysed.

### *Assays*

The assays for PIIINP, PICP and ICTP have previously been reported in detail (Melkko *et al.*, 1990; Risteli *et al.*, 1988, 1993). The reference intervals among healthy Finnish blood donors over 20 years of age are as follows: PIIINP 1.7–4.3 $\mu g\ l^{-1}$ (Risteli *et al.*, 1988), PICP 50–170 $\mu g\ l^{-1}$ for women and 40–200 $\mu g\ l^{-1}$ for men (Melkko *et al.*, 1990) and ICTP 1.7–4.6 $\mu g\ l^{-1}$ (Risteli *et al.*, 1993). For patients less than 20 years old the upper limit of the reference range is higher, particularly for ICTP in young men (about 11 $\mu g\ l^{-1}$ in men 16–18 years old, and about 9 $\mu g\ l^{-1}$ in men 18–20 years old) (P Trivedi and J Risteli, unpublished data). The intra- and interassay coefficient of variation for the assays are around 5%.

### *Statistical analysis*

The changes in collagen metabolites within the patients were studied with the Wilcoxon signed-rank test. Correlations between the different metabolites, and between the metabolites and age were assessed by the Spearman's rank-order correlation coefficient ($r_s$). The effect on different variables on survival was studied with the method of proportional hazards. In these analyses the log transformed values of the metabolites were used as continuous variables.

### Results

### *Pretreatment values*

The pretreatment concentrations of PIIINP, PICP and ICTP are shown in Table I. There is no difference between the concentrations obtained for those patients who later remained in continuous remission and those with a later relapse of the disease. For alkaline phosphatase (AP) and lactate

**Table I** The mean baseline (= before the start of the preoperative chemotherapy) values, range and proportion above the reference range of PIIINP, PICP and ICTP in all MO patients, and separately for patients subsequently in continuous remission or who have had a relapse

| *Reference range* | *PIIINP (µg l⁻¹) 1.7–4.3* | *PICP (µg l⁻¹) 50–170 (women) 40–200 (men)* | *ICTP (µg l⁻¹) 1.7–4.6* |
|---|---|---|---|
| All patients ($n$ = 18) | | | |
| Mean | 5.3 | 134 | 9.4 |
| Range | 3–11.8 | 73–267 | 2.5–18.3 |
| Proportion above reference range | 61% | 17% | 78% |
| Continuous remission ($n$ = 8) | | | |
| Mean | 4.6 | 134 | 8.5 |
| Range | 3.2–6 | 73–267 | 3.1–17 |
| Proportion above reference range | 63% | 25% | 63% |
| Relapse ($n$ = 10) | | | |
| Mean | 5.8 | 133 | 10.2 |
| Range | 3–11.8 | 75–195 | 2.5–18.3 |
| Proportion above reference range | 60% | 10% | 90% |

PIIINP, the amino-terminal propeptide of type III procollagen; PICP, the carboxy-terminal propeptide of type I procollagen; ICTP, the mature, pyridoline or pyrrole cross-linked carboxy-terminal telopeptide of type I collagen.

dehydrogenase (LD) the mean pretreatment activities were 585 U $l^{-1}$ (range 115–2178) and 405 U $l^{-1}$ (261–721) respectively.

There was a statistically significant positive correlation between the baseline PIIINP and PICP ($r_s$ = 0.54, $P$ = 0.02), between PIIINP and ICTP ($r_s$ = 0.49, $P$ = 0.04), but not between PICP and ICTP or between the collagen metabolites and AP or LD. In addition there was a statistically significant negative correlation between age and ICTP ($r_s$ = −0.68, $P$ = 0.005), but not between age and other collagen metabolites, or AP or LD and age.
None of the collagen metabolites were prognostic factors for survival at baseline. However, AP and LD were significant prognostic factors for both overall and metastases-free survival ($P$ = 0.04 and 0.03 respectively).

*Metastatic disease*

In metastatic disease the mean PIIINP was 4.4 µg $l^{-1}$ (2.7–6.9 µg $l^{-1}$, 60% above reference range), PICP 107 µg $l^{-1}$ (36–200 µg $l^{-1}$, 20%), and ICTP 7.9 µg $l^{-1}$ (3.2–22.3 µg $l^{-1}$, 67%). In patients with both baseline values and values at time of metastases available these did not differ significantly. There was no rising level of the metabolites preceding the detection of metastases (Figure 1a–c).

*Effect of the treatment*

All the metabolites decreased from baseline during the preoperative chemotherapy. This decrease was statistically significant for PIIINP and PICP ($P$ = 0.009 and 0.004 respectively). The mean change was −1.2 µg $l^{-1}$ and −44.5 µg $l^{-1}$, respectively, and samples were obtained before surgery a mean of 37 days after the start of the chemotherapy (range 14–77 days). Post-operatively ICTP was significantly above the baseline ($P$ = 0.04), the mean change was 2.3 µg $l^{-1}$, samples taken a mean of 16 days post-operatively (range 4–34 days). PIIINP and PICP also increased post-operatively (changes not significant). By the time of the last chemotherapy cycle (samples taken a mean of 176 days post-operatively, range 77–314 days) both PIIINP and ICTP were marginally above, and PICP marginally below the pretreatment level. These changes were statistically significant only for PICP ($P$ = 0.03). The changes in the levels of PIIINP, PICP and ICTP relative to baseline during the therapy and the follow-up are shown in Figure 2a–c. There was considerable interpatient variability in the changes in the levels of the collagen metabolites after the completion of the chemotherapy. The mean ICTP was within the

**Figure 1** Changes in PIIINP (**a**), PICP (**b**) and ICTP (**c**) preceding detection of metastases. Time = 0 indicates the date of clinical diagnosis of metastases. The metabolites are indicated as relative values (i.e. the actual value divided by the value obtained at time of metastases). Thus the value obtained at time of metastases by definition = 1.

reference range within 5 years, and PIIINP within 8 months after the last chemotherapy infusion. The changes in PICP were at most a slight increase of short duration after the completion of the post-operative chemotherapy.

**Figure 2** The changes in PIIINP (**a**), PICP (**b**) and ICTP (**c**) during the therapy and the follow-up. A, baseline value; B, preoperative value; C, post-operative value; D, value during last post-operative chemotherapy cycle; 1 month, values obtained 1 month after the last chemotherapy infusion, and thereafter monthly up to 1 year after the last chemotherapy cycle (= 1 year). From this point values are shown yearly until 10 years. Only measurements obtained during disease-free follow-up are included (i.e. from date of metastases all measurements are excluded). Bottom, individual graphs for each patient. Top, mean and 95% confidence intervals (at 7 months and 12 months after completion of therapy less than four measurements were available, these were excluded).

### Discussion

The treatment results on osteosarcoma have greatly improved during recent decades (Link *et al.*, 1991). There is however a need for intensified treatment in selected patients, and thus also for new sensitive and specific prognostic factors.

Based on our experiences on soft tissue sarcomas and multiple myeloma as well as on prostate cancer metastatic to bone we had reasons to expect that metabolites of type I and III collagen could be of use in osteosarcoma (Elomaa *et al.*, 1992; Taube *et al.*, 1992; Wiklund *et al.*, 1992; Kylmälä *et al.*, 1993).

The present study indicates that elevated concentrations of type I and III collagen metabolites, particularly ICTP and PIIINP, are frequent findings in untreated osteogenic sarcoma. On the other hand, the circulating concentrations of PICP, which as part of the type I procollagen is a characteristic gene product of the osteoblastic cells, was much less often increased, suggesting that the malignant cells are not more active in producing this protein than are the normal osteoblasts. AP is another product of the osteoblast, and considered to be expressed later than type I collagen in the phenotypic development of this cell (Stein *et al.*, 1990). It is interesting that this enzyme is a prognostic factor with respect to survival in osteosarcoma, a finding that was confirmed here, as also the prognostic implication of LD (Link *et al.*, 1991; Bacci *et al.*, 1993).

We report two important findings here. Firstly, in contrast to previous studies on these metabolites in malignant disease our study population was made up almost exclusively of young persons. The majority of patients had initially elevated values of PIIINP and ICTP. It is probable that part of this elevation is a result of the young age of the patients (P Trivedi, unpublished data). Furthermore, the correlation between ICTP and age suggests this.

Secondly, this study confirms our previous findings on the effect of therapy *per se* on collagen metabolism (Haukipuro *et al.*, 1990; Wiklund *et al.*, 1993). The intensive multimodality treatment of osteosarcoma clearly affect the collagen metabolism. In this study population the long disease-free follow-up made it possible to analyse the time it takes for collagen metabolism to normalise. For ICTP the time is surprisingly long. However, although the pattern of decline was similar in patients above or below 20 years (data not shown) it should be emphasised that all patients except one were below 35 years of age at diagnosis, and thus the type I collagen metabolism could be expected to be more active than in an older age. Thirdly, these findings emphasise that clinical results from biochemical markers reflecting tissue destruction and repair, such as the three used here, must be interpreted with caution.

**Acknowledgements**

The study was financially supported by a grant from Finska Läkaresällskapet (Finnish Medical Society), the Finnish Cancer Foundations and the Medical Research Council of the Academy of Finland.

### References

ABILDGAARD N, NIELSEN JL AND HEICKENDORFF L. (1994). Connective tissue components in serum in multiple myeloma: analyses of propeptides of type I and type III procollagens, type I collagen telopeptide and hyaluronan. *Am. J. Hematol.*, **46,** 173–178.

BACCI G, PICCI P, FERRARI S, ORLANDI M, RUGGIER P, CASADEI R, FERRARO A, BIAGINI R AND BATTISTINI A. (1993). Prognostic significance of serum alkaline phosphatase measurements in patients with osteosarcoma treated with adjuvant or neoadjuvant chemotherapy. *Cancer*, **71,** 1224–1230.

ELOMAA I, VIRKKUNEN P, RISTELI L AND RISTELI J. (1992). Serum concentration of the cross-linked carboxyterminal telopeptide of type I collagen (ICTP) is a useful prognostic indicator in multiple myeloma. *Br. J. Cancer*, **66,** 337–341.

HAUKIPURO K, RISTELI L, KAIRALUOMA MI AND RISTELI J. (1990). Aminoterminal propeptide of type III procollagen in serum during wound healing in human beings. *Surgery*, **107,** 381–388.

KYLMÄLÄ T, TAMMELA T, RISTELI L, RISTELI J, TAUBE T AND ELOMAA I. (1993). Evaluation of the effect of oral clodronate on skeletal metastases with type 1 collagen metabolites. A controlled trial of the Finnish Prostate Cancer Group. *Eur. J. Cancer*, **29A,** 821–825.

KYLMÄLÄ T, TAMMELA TLJ, RISTELI L, RISTELI J, KONTTURI M AND ELOMAA I. (1995). Type I collagen degradation product (ICTP) gives information about the nature of bone metastases and has prognostic value in prostate cancer. *Br. J. Cancer*, **71,** 1061–1064.

LINK MP, GOORIN AM, HOOWITZ M, MEYER WH, BELASCO J, BAKER A, AYALA A AND SHUSTER J. (1991). Adjuvant chemotherapy of high-grade osteosarcoma of the extremity. Updated results of the multi-institutional osteosarcoma study. *Clin. Orthop.*, **270,** 8–14.

MELKKO J, NIEMI S, RISTELI L AND RISTELI J. (1990). Radioimmunoassay of the carboxyterminal propeptide of human type I procollagen. *Clin. Chem.*, **36,** 1328–1332.

RISTELI J, ELOMAA I, NIEMI S, NOVAMO A AND RISTELI L. (1993). Radioimmunoassay for the pyridinoline cross-linked carboxyterminal telopeptide of type I collagen: a new serum marker of bone collagen degradation. *Clin., Chem.*, **39,** 635–640.

RISTELI J, NIEMI S, TRIVEDI P, MAENTAUSTA O, MOWAT AP AND RISTELI L. (1988). Rapid equilibrium radioimmunoassay for the amino-terminal propeptide of human type III procollagen. *Clin. Chem.*, **34,** 715–718.

RISTELI L AND RISTELI J. (1989). Noninvasive methods for detection of organ fibrosis. In *Connective tissue in Health and Disease*, Rojkind M. (ed.) pp. 61–98. CRC Press: Boca Raton, Florida.

STEIN GS, LIAN JB AND OWEN TA. (1990). Relationship of cell growth to the regulation of tissue-specific gene expression during osteoblast differentiation. *FASEB J.*, **4,** 3111–3123.

TAUBE T, FRANSSILA K, RISTELI L, RISTELI J AND ELOMAA I. (1992). Monitoring of multiple myeloma and bone marrow fibrosis with aminoterminal propeptide of type III collagen (PIIINP). *Br. J. Haematol.*, **82,** 32–37.

WIKLUND TA, ELOMAA I, BLOMQVIST CP, RISTELI L AND RISTELI J. (1992). Type III collagen metabolism in soft tissue sarcomas. *Br. J. Cancer*, **65,** 193–196.

WIKLUND TA, BLOMQVIST CP, RISTELI L, RISTELI J AND ELOMAA I. (1993). Impact of chemotherapy on collagen metabolism: a study of serum PIIINP (aminoterminal propeptide of type III procollagen) in advanced sarcomas. *J. Cancer. Res. Clin. Oncol.*, **119,** 160–164.